# Enhanced Citric Acid Production through *Aspergillus niger*: Insights from Fermentation Studies Using Sugarcane Molasses

**DOI:** 10.3390/life14060756

**Published:** 2024-06-13

**Authors:** Samina Khurshid, Hamad Ashraf, Tanveer Hussain, Muhammad Iqbal, Huma Qureshi, Tauseef Anwar, Saleh H. Salmen, Mohammad Javed Ansari

**Affiliations:** 1 Department of Biotechnology, Virtual University (Lahore Campus), Lahore 54000, Pakistan; 2 Department of Botany, Government Dyal Singh Graduate College, Lahore 54000, Pakistan; 3 Department of Botany, University of Chakwal, Chakwal 48800, Pakistan; 4 Department of Botany, The Islamia University of Bahawalpur, Bahawalpur 63100, Pakistan; 5 Department of Botany and Microbiology, College of Science, King Saud University, Riyadh 11451, Saudi Arabia; 6 Department of Botany, Hindu College Moradabad (Mahatma Jyotiba Phule Rohilkhand University Bareilly), Moradabad 244001, India

**Keywords:** citric acid, submerged fermentation, sugar cane molasses, industrial applications

## Abstract

The production of citric acid, a vital agricultural commodity utilized across various industries such as food, beverages, pharmaceuticals, agriculture, detergents, and cosmetics, predominantly relies on microbial fermentation, with *Aspergillus niger* accounting for approximately 90% of global production. In this study, we aimed to optimize the key factors influencing citric acid production, with a focus on strains, fermentation techniques, and carbon sources, particularly sugarcane molasses. *A. niger*, sourced from the Botany department/Biotechnology laboratories at Govt. College of Science, Lahore, was employed for citric acid production. The process involved inoculum preparation through spore collection from 3 to 5 days of cultured PDA slants. The fermentation medium, comprising cane molasses with a 15% sugar concentration, was meticulously prepared and optimized for various factors, including magnesium sulfate, potassium ferrocyanide, time of addition of potassium ferrocyanide, ammonium oxalate, and calcium chloride. Our optimization results shed light on the significant impact of different factors on citric acid production. For instance, the addition of 0.4 g/L magnesium sulfate led to a maximum yield of 75%, while 2 g/L potassium ferrocyanide, added at 24 h, achieved a yield of 78%. Remarkably, ammonium oxalate, at a concentration of 10 g/L, resulted in a notable 77% yield. Conversely, the addition of calcium chloride exhibited negligible effects on citric acid production, with the control group yielding more at 78%. Our study underscores the potential for optimizing factors to enhance citric acid production by *A. niger* in submerged fermentation. These findings highlight the pivotal role of magnesium sulfate, potassium ferrocyanide, and ammonium oxalate in augmenting citric acid yields while emphasizing the minimal impact of calcium chloride. Ultimately, these insights contribute to advancing our understanding of microbial citric acid biosynthesis, providing valuable implications for industrial applications and future research endeavors.

## 1. Introduction

Citric acid is an essential primary agricultural product that is extensively used in the world. It acts as an intermediate in the tricarboxylic acid cycle (citric acid cycle) during the oxidation of carbohydrates to carbon dioxide [1,2]. Citric acid has an important role in industries involving the production of food products, beverages, pharmaceutical and agricultural products, detergents, and cosmetics. The production of citric acid, a crucial agricultural commodity with widespread applications in various industries including food, beverages, pharmaceuticals, agriculture, detergents, and cosmetics, heavily relies on microbial fermentation, predominantly employing *Aspergillus niger*, which accounts for approximately 90% of global production. Several major companies lead the industrial-scale production of citric acid, including ADM (Archer Daniels Midland), Cargill, Tate & Lyle, Jungbunzlauer, and Weifang Ensign Industry Co., Ltd. These companies operate fermentation facilities across multiple regions, with annual production capacities reaching hundreds of thousands of metric tons. Through their extensive operations, they cater to the diverse demands of global markets, ensuring a steady and substantial supply of citric acid for various applications. This method is a stable, maintainable (because of its lesser consumption of energy), simple (because of the ease of its approaches), and economical process for obtaining citric acid [3,4]. Citric acid is produced by three fermentation techniques, namely, surface culture technique, submerged culture technique, and solid-state fermentation. The production of citric acid by *Aspergillus niger* through submerged fermentation appears to be a highly desirable and most widely used technique for the microbial production of citric acid [4,5].

A successful process depends on an appropriate strain and optimization of fermentation factors. Different microorganisms are involved in citric acid fermentation, such as bacteria, fungi, and yeast, but *Aspergillus niger* has a profound role in the large-scale production of citric acid [6]. *A. niger* has become the organism of choice for the maximum yield of citric acid [7]. The main advantages of using *A. niger* are its ease of handling and its abilities to ferment a variety of cheap raw materials and deliver high yields. Citric acid is the primary product of *A. niger’s* metabolism due to its well-developed homologous and heterologous extracellular enzymatic system [3].

Some sources mainly used as a carbon source for citric acid production are sugar cane syrup, sugar cane molasses, sugar beet molasses, sugar cane bagasse, sweet potatoes, apple peels, and grape pomace. Cane molasses is an ideal medium for citric acid production by microbial fermentation owing to its easy availability and low-cost value. It is a byproduct of the sugar industry containing 40–60% carbohydrates and acts as a useful carbon source for microbial growth and citric acid production. However, it has certain compounds, such as metal ions, that inhibit the process of citric acid production. Factors such as the optimum level of carbon and nitrogen, metal salts, and phosphate concentration in the medium were found to influence citric acid production [8]. Different methods have been employed to eliminate the inhibitory metallic substances from the cane molasses. Molasses was treated with different concentrations of K_4_[Fe(CN)_6_] for the removal of undesirable metal ions and prevention of their inhibitory effects on the iso-citric dehydrogenase [6]. Potassium ferrocyanide accelerates the catalytic activity of condensing enzymes of citric acid by blocking the poisonous effect of some metal ions containing iron, zinc, copper, magnesium, calcium, potassium, and sodium. The high concentrations of Fe^2+^ and Mn^2+^ have an inhibitory effect on citric acid fermentation. The dilution of molasses to 15% sugar concentration and the addition of K_4_[Fe(CN)_6_] help in diluting the undesirable amount of trace elements [9]. The metal ions Fe^2+^ and Zn^2+^ have a major role in citric acid production at low concentrations because their higher concentration exhibits the inhibitory effect on mycelial growth and citric acid extraction [9,10].

Cane molasses has a high ratio of calcium, which has an inhibitory effect on the mycelial growth of fungus, so ammonium oxalate is added to the medium to inhibit the extra amount of calcium [10]. Magnesium sulfate is very important for the catalytic activity of various enzymes for the growth of *A. niger* and citric acid production from microbial cells. Thus, the addition of magnesium sulfate not only provides the magnesium and sulfate ions to the medium for enzyme activation but also enhances citric acid production [9,10]. Calcium chloride in a fermentation medium greatly promotes mycelium growth with a large number of pellets, which stimulate the lytic enzymes that have an inhibitory effect on citric acid production [11]. Other factors also influence the yield of citric acid. These include the amount of nutrients, environmental factors, pH range, and size of inoculum used by varying the number of spores added as inoculum in the culture medium. These should be maintained at an optimum level according to the availability of the substrate. Oxygen supply to the culture medium is also a very critical factor for the maximum microbial growth to obtain a sufficient yield of citric acid with the controlled condition of the experiment [12,13].

In the present study, different factors (various concentrations of MgSO_4_·7H_2_O, K_4_[Fe(CN)_6_], time of addition of K_4_[Fe(CN)_6_], ammonium oxalate and CaCl_2_·2H_2_O) affecting citric acid production from sugar cane molasses by *A. niger* through submerged fermentation will be studied.

## 2. Materials and Methods

A strain of *Aspergillus niger* was used in the experimental work for citric acid production. The strain of *A. niger* was collected from the culture collection of the Botany department/ Biotechnology laboratories, Govt. College of Science Wahdat Road Lahore.

### 2.1. Inoculum Preparation

Spores from 3 to 5 days culture from PDA slants were scraped after adding 5 mL of sterilized saline solution containing 8 g NaCl/L. The test tubes were shaken vigorously with glass beads to break clumps of conidia. Spore count was performed by direct hemocytometer counting slide.

### 2.2. Fermentation Medium

Medium-containing sugarcane molasses with 15% sugar concentration were prepared. For this purpose, molasses was clarified and diluted to about 25% sugar level in which 35 mL of 0.1 N H_2_SO_4_ was poured, and the mixture was boiled for ½ h, then cooled and neutralized by adding Ca (OH)_2_ at pH 6.0.

The mixture was allowed to stand overnight. A clear supernatant (273 g/L) of cane molasses was diluted with distilled water up to 1 L to achieve a 15% total sugar concentration. Moreover, 1 liter of this clear supernatant-diluted molasses, including urea 1.2 g/L, magnesium sulfate 0.4 g/L, H_3_PO_4_ 0.4 g/L, and pH 6.0, was used as culture medium. For submerged fermentation, 100 mL of fermentation medium was added in 250 mL of conical flask, cotton plugged, and sterilized in an autoclave at 121 °C for 15 min with 15 lbs/inch^2^ of pressure.

### 2.3. Optimization of Factors

A factorial experimental design was conducted to optimize the factors affecting citric acid production. Each treatment was performed in triplicate to ensure the accuracy and reproducibility of the results.

#### 2.3.1. Magnesium Sulfate (MgSO_4_·7H_2_O)

Various amounts of MgSO_4_.7H_2_O from (0.0 to 4.0 g/L) were added in 100 mL of culture medium containing inoculum volume (5 × 10^6^ spores/100 mL) in each flask, respectively, and kept on a rotary shaker for 6 days at 30 °C. After, fermentation media was evaluated, and the optimum magnesium sulfate concentration was determined.

#### 2.3.2. Potassium Ferrocyanide

Culture media containing 100 mL, with pH of 6, optimized inoculum volume, and various potassium ferrocyanide concentration ranges (0.20 to 3.0 g/L) were taken in flasks, respectively, and placed on a rotary shaker for 6 days at 30 °C, and optimum potassium ferrocyanide concentration was nominated.

#### 2.3.3. Time of Addition of Potassium Ferrocyanide

The optimum time for the addition of potassium ferrocyanide was detected by adding the 2.0 g/L (optimized concentration) of K_4_[Fe(CN)_6_] at different time intervals from 20 to 168 h in sterilized culture media with pH of 6 and optimal inoculum volume and put it on a rotary shaker for 6 days at 30 °C.

#### 2.3.4. Ammonium Oxalate

Flasks having 5 to 25 g/L concentrations of ammonium oxalate, 100 mL sterilized fermentation medium of pH of 6, and optimized inoculum volume, respectively, were laid in a rotary shaker for 6 days at 30 °C, and the maximum concentration of ammonium oxalate was determined, showing maximum C.A production.

#### 2.3.5. Calcium Chloride (CaCl_2_·2H_2_O)

The same conditions were applied to find the maximum concentration of calcium chloride by taking the various concentrations of calcium chloride (0.0 to 1.40 g/L), sterilized fermentation media (100 mL) of pH of 6, and optimized inoculum volume.

### 2.4. Analysis

#### 2.4.1. Mycelium Weight

D.M.W was estimated and filtered the culture medium by using weighed Whatman filter paper 44. Mycelium was washed with distilled water and dried at 110 °C overnight until constant weight was attained.

#### 2.4.2. Reducing Sugar Infiltrate

The sugar concentration was measured calorimetrically using the DNS (3, 5 Dinitro salicylic acid) method. For this, 2 mL DNS was added to 2 mL filtrate and heated for 5 min. The mixture was cooled, and final volume was raised to 20 mL using distilled water. This solution was checked via colorimeter for estimation of sugar concentration at 530 nm and compared and checked by the standard curve of glucose.

#### 2.4.3. Estimation of Citric Acid by Titration Method

The citric acid was measured using titration method. The filtrate was titrated with 0.1 N NaOH. Phenolphthalein was used as an indicator. The appearance of pink color indicated the endpoint. The amount of produced citric acid was determined in terms of normality using the material balance equation and later was converted into g/L as reported by Anand and Venkat 2012.

N1V1=N2V2

$$\%age\;total\;acid=\frac{Titre\times normality\;of\;NaOH\times equivalent\;weight\;of\;citric\;acid}{Volume\;of\;sample\times 1000}\times 100$$

The percentage yield of citric acid was calculated by sugar consumed as follows:

$$\%Yield\;of\;Citric\;Acid=\frac{Citric\;Acid}{Sugar\;used}\times 100$$

### 2.5. Statistical Analysis

Data were analyzed using Analysis of Variance (ANOVA) to evaluate the significance of the different factors and their interactions with citric acid production. Results were expressed as means ± standard deviations. Statistical significance was considered at *p* < 0.05.

## 3. Results and Discussion

### 3.1. Magnesium Sulfate (MgSO_4_·7H_2_O)

Various concentrations of magnesium sulfate were explored during our experimental work on the yield of citric acid. For this purpose, magnesium sulfate was added to a fermentation medium ranging from 0.0 to 4.0 g/L. A result was obtained after the addition of various concentrations of magnesium sulfate. The percentage yield of citric acid was increased by increasing the amount of MgSO_4_·7H_2_O. The extreme amount of C. A was obtained at 0.4 g/L of magnesium sulfate by adding in the fermentation medium. These results are represented in Table 1, which explains the extent of citric acid production with different concentrations of magnesium sulfate. In the graph, a maximum citric acid yield of 75%, D.M.W 38 g/L, 99.5 g/L of sugar consumed obtained under 0.4 g/L of MgSO_4_·7H_2_O can be seen. Therefore, a 0.4 g/L magnesium sulfate concentration is proven to be optimum for further experimentation.

#### 3.1.1. Potassium Ferrocyanide

The result showed the effect of various concentrations of potassium ferrocyanide (0.0 to 3.0 g/L) on citric acid synthesis by *Aspergillus niger*. The low amount of citric acid recorded was by the control medium, but by the addition of K_4_[Fe(CN)_6_], the production of citric acid reached its maximum level. A maximum yield of C. A % age, dry mycelial weight (D.M.W), and sugar consumed were 78%, 38 g/L, and 99 g/L. The results gained from various concentrations of potassium ferrocyanide were expressed in Table 2. The graph was plotted between different concentrations of potassium ferrocyanide and the percentage yield of citric acid, sugar consumed, and D.M.W. The graph showed that the highest ranges of citric acid yield, sugar consumed, and D.M.W were recorded with 2.0 g/L potassium ferrocyanide in the medium. An increase in the concentration of potassium ferrocyanide proved toxic and caused a reduction in the yield of citric acid.

#### 3.1.2. Time of Addition of K_4_[Fe(CN)_6_]

The optimum percentage yield was 79% of citric acid, 99.5 g/L sugar consumed, and 32 g/L D.M.W amounts were observed by adding K_4_[Fe(CN)_6_] at 24 h (after spores’ inoculation). A further increase or decrease in the time addition of potassium ferrocyanide greatly reduced the citric acid yield. The results obtained after fermentation were expressed with the help of Table 3, which was plotted between the times of addition of potassium ferrocyanide ranging from 24 to 168 h against citric acid percentage yield, sugar consumed, and D.M.W.

#### 3.1.3. Ammonium Oxalate

The ammonium oxalate ranged from 0.00 to 40.00 g/L, dissolved in molasses medium, and results are expressed in Table 4. The readings on the graph showed that the maximum percentage yield of citric acid was 78%, sugar consumed at 98.5 g/L, and D.M.W at 33 g/L with the addition of 10 g/L of ammonium oxalate. The further change in the amount of ammonium oxalate in the molasses medium proved inhibitory for the process of citric acid production.

#### 3.1.4. Calcium Chloride (CaCl_2_·2H_2_O)

Calcium chloride was added in eight different concentrations ranging from 0.0 g/L to 1.40 g/L. The result obtained from the experiment by using different concentrations of ammonium oxalate was expressed with the help of Table 5, plotted against the percentage yield of citric acid, sugar consumed, and D.M.W. It was observed that the optimum yield of citric acid was 78%, sugar consumed was 99 g/L, and D.M.W was 35 g/L without the addition of calcium chloride. The quantity of citric acid abruptly reduced with the addition of CaCl_2_·2H_2_O.

The utilization of agro-industrial byproducts, such as sugarcane molasses, as substrates for microbial fermentation has garnered significant attention due to their abundance and potential for value-added product synthesis [14]. *Aspergillus niger* is well-known for its ability to efficiently degrade such substrates and secrete various organic acids, including citric acid, which holds paramount importance in the food industry as an additive, preservative, and antimicrobial agent against spoilage [15].

In this study, we focused on evaluating key factors influencing citric acid (C.A) production by *A. niger*. Notably, we achieved a substantial increase in the percentage yield of citric acid (78%) by carefully manipulating variables such as magnesium sulfate, potassium ferrocyanide, and ammonium oxalate concentrations, as well as the timing of their addition during the fermentation process. Our findings align well with the existing literature, where the addition of magnesium sulfate (MgSO_4_·7H_2_O) at a concentration of 0.4 g/L has been reported to enhance citric acid yield (75%). This augmentation can be attributed to the provision of essential Mg^2+^ and (SO_4_)^2−^ ions, which play pivotal roles in enzymatic activation, metabolic processes, fungal growth, and, ultimately, citric acid production. Consistently, previous studies by El-Kady, Fomina et al., and Munir et al. have emphasized the positive impact of magnesium sulfate on citric acid synthesis in *Aspergillus niger* [10,16,17]. However, it is noteworthy that higher concentrations of MgSO_4_·7H_2_O have been associated with inhibitory effects on citric acid accumulation and fungal enzymatic activity [18]. This suggests a delicate balance in optimizing magnesium sulfate concentrations to maximize citric acid production without impeding fungal growth and metabolic activity.

Potassium ferrocyanide was also used in the sugarcane molasses (fermentation medium) to obtain the maximum accumulation of C.A. The citrate concentration was reached at a higher level by increasing the concentration of K_4_[Fe(CN)_6_] as compared to untreated molasses, which was quite low in comparison. This outcome was related to research work carried out by [9,10,19]. Potassium ferrocyanide was added to the fermentation medium to precipitate the heavy metals and prevent their inhibitory effect on citric acid production. This finding is derived from work reported by El-Kady and Hauka et al. [9,10]. In this recent work, an extreme accumulation of C. A (78%) was achieved with a 2.0 g/L potassium ferrocyanide addition in the fermentation medium at 24 h after spore inoculation. A further increase or decrease in the instances of the addition of K_4_[Fe(CN)_6_] directly affects the growth and productivity level. The sensitivity of the fermentation medium to K_4_[Fe(CN)_6_] was reported by Kareem [20].

Ammonium oxalate also has a great role in enhancing the percentage yield of citric acid. A concentration of 10 g/L of ammonium oxalate was added in cane molasses, which rapidly precipitated the undesirable calcium oxalate and calcium to unavailable form for maximum yield (77%) of citric acid. The findings from various studies on the role of calcium ions and other factors in fermentation processes align closely with our results, highlighting the critical influence of calcium on both ethanol and citric acid production [9,10,21,22]. In our study, calcium residues in molasses significantly decreased the efficiency of ethanol fermentation by *Saccharomyces cerevisiae*, with higher concentrations leading to greater inhibition. This mirrors findings from high-yield ethanol production studies, where high concentrations of K^+^ and Ca^2+^ were identified as primary limiting factors. Similarly, in citric acid production by *Aspergillus niger*, the addition of ammonium oxalate to precipitate calcium as calcium oxalate greatly enhanced citric acid yield, supporting our observation that calcium management is essential. These consistent observations across different studies emphasize the importance of controlling calcium and other metal ions to optimize fermentation efficiency and product yields.

During the current research, the effect of CaCl_2_ on the yield of citric acid was investigated. The result of this study proved that calcium chloride has no impact on the yield as the control medium showed a higher % age yield of citric acid (78%) as compared to the addition of calcium chloride in the fermentation medium [7,9,23]. This might be due to the production of lytic enzymes and oxalate in the medium. These findings highlight the potential to improve citric acid yield by optimizing cytosolic pathways, complementing the industrial application of *Aspergillus niger* for enhanced citric acid production [24]. These results emphasize the importance of optimizing fermentation conditions, such as cytosolic pathways, to enhance citric acid production by *A. niger* [25], aligning with our work’s focus on improving citric acid yield using sugarcane molasses.

## 4. Conclusions

This study successfully explored various factors influencing citric acid production by *Aspergillus niger* utilizing sugarcane molasses as a substrate. Through systematic experimentation, it was found that the addition of 0.4 g/L of magnesium sulfate, 2 g/L of potassium ferrocyanide, and 10 g/L of ammonium oxalate significantly enhanced citric acid production, reaching a maximum yield of 78%. Moreover, the timing of the addition of potassium ferrocyanide was critical, with the optimal time being 24 h after spore inoculation. Interestingly, calcium chloride did not show any significant impact on citric acid yield. These findings contribute to the understanding of the factors influencing citric acid production and offer insights into optimizing the process for industrial applications. Additionally, the results align with previous research, validating the efficacy of the optimized conditions in enhancing citric acid production. Further studies could explore additional parameters to refine the production process and improve overall efficiency.

## Figures and Tables

**Table 1:** Optimization of MgSO_4_·7H_2_O concentration for citric acid production by *Aspergillus niger*.

MgSO_4_·7H_2_O (g/L)	D.M.W (g/L) ± SD	Sugar Consumed (g/L) ± SD	Citric Acid Produced (g/L) ± SD	% Yield
0.00	15.00 ± 2.56 b	50.00 ± 8.66 b	15.00 ± 4.24 b	30 ± 7.48 b
0.10	16.00 ± 2.56 b	55.00 ± 7.35 b	20.00 ± 5.66 bc	36 ± 6.56 bc
0.20	16.50 ± 2.08 b	60.50 ± 6.54 c	26.00 ± 4.24 c	42 ± 6.78 cd
0.30	18.50 ± 4.36 bc	68.00 ± 9.54 d	32.50 ± 7.42 d	47 ± 8.21 de
0.40	38.00 ± 6.32 ef	99.50 ± 11.55 i	75.00 ± 14.85 h	75 ± 9.85 f
0.50	26.00 ± 4.58 d	90.50 ± 8.76 h	55.00 ± 10.20 g	60 ± 7.65 ef
0.60	24.00 ± 3.46 d	85.00 ± 7.21 g	48.50 ± 9.15 f	56 ± 6.98 efg
0.70	20.50 ± 3.08 c	79.50 ± 5.46 f	42.00 ± 7.07 e	52 ± 5.98 def
0.80	18.50 ± 2.56 bc	72.50 ± 4.58 e	35.70 ± 6.22 d	48 ± 5.86 def
0.90	18.00 ± 2.08 bc	65.00 ± 3.61 cd	28.00 ± 4.58 cd	43 ± 4.98 def
1.00	17.00 ± 2.56 bc	58.50 ± 3.46 bc	23.50 ± 3.61 bc	39 ± 4.21 def
2.00	16.50 ± 2.08 b	55.00 ± 3.08 b	20.00 ± 2.83 bc	36 ± 3.46 bc
3.00	15.00 ± 2.56 b	42.00 ± 2.56 a	14.50 ± 2.56 b	34 ± 2.56 bc
4.00	12.00 ± 1.73 a	40.00 ± 2.08 a	10.70 ± 1.73 a	26 ± 1.87 a

Means with different letters within a column are significantly different (*p* < 0.05).

**Table 2:** Optimization of potassium ferrocyanide concentration for citric acid production by *Aspergillus niger*.

K_4_Fe(CN)_6_ (g/L)	D.M.W (g/L)	Sugar Consumed (g/L)	Citric Acid (g/L)	% Yield
0.00	10.5 ± 1.45 a	25.00 ± 2.65 a	3.36 ± 0.76 a	13 ± 3.46 a
0.20	12.00 ± 1.87 ab	28.00 ± 3.08 ab	4.50 ± 1.22 ab	16 ± 3.98 ab
0.40	15.00 ± 2.08 bc	30.50 ± 3.46 bc	5.90 ± 1.73 bc	19 ± 4.21 bc
0.60	20.50 ± 3.46 cd	42.00 ± 4.58 cd	15.00 ± 3.46 cd	35 ± 5.98 cd
0.80	22.60 ± 3.98 de	55.00 ± 5.46 de	20.50 ± 4.58 de	36 ± 6.32 cd
1.20	25.00 ± 4.36 ef	60.50 ± 6.32 ef	25.00 ± 5.66 ef	41 ± 6.78 e
1.50	30.00 ± 5.00 fg	85.00 ± 7.21 h	45.00 ± 6.71 gh	52 ± 7.35 f
2.00	38.00 ± 6.16 gh	99.00 ± 8.66 i	78.00 ± 9.54 i	78 ± 8.98 h
2.50	26.00 ± 4.58 ef	75.00 ± 6.78 g	42.00 ± 7.21 h	56 ± 7.65 g
3.00	22.00 ± 3.74 de	63.00 ± 5.00 ef	28.00 ± 4.36 fg	44 ± 6.00 ef

Means with different letters within a column are significantly different (*p* < 0.05).

**Table 3:** Optimization of time of addition of potassium ferrocyanide concentration for citric acid production by *Aspergillus niger*.

Time of Addition (h)	Concentration of Potassium Ferrocyanide (g/L)	D.M.W (g/L)	Sugar Consumed (g/L)	Citric Acid Produced (g/L)	% Yield
0	2.00	28.00 ± 4.58 d	90 ± 8.66 f	50 ± 7.07 de	61 ± 7.81 c
24	2.00	32.00 ± 5.00 e	99.50 ± 9.54 g	77 ± 8.66 f	77 ± 8.79 e
48	2.00	26.00 ± 4.36 cd	85 ± 7.21 e	58 ± 6.78 e	64 ± 7.21 d
72	2.00	25.00 ± 4.00 cd	80 ± 6.78 d	48 ± 5.66 d	60 ± 6.32 c
96	2.00	22.00 ± 3.74 c	75 ± 6.32 c	42 ± 5.29 c	56 ± 5.98 bc
120	2.00	20.50 ± 3.46 b	71 ± 5.29 b	38 ± 4.69 bc	53 ± 5.00 b
144	2.00	18.50 ± 2.87 ab	65 ± 4.58 ab	28 ± 3.46 a	43 ± 4.12 ab
168	2.00	17.00 ± 2.45 a	60 ± 3.87 a	35 ± 4.24 b	41 ± 3.98 a

Means with different letters within a column are significantly different (*p* < 0.05).

**Table 4:** Optimization of ammonium oxalate concentration for citric acid production by *Aspergillus niger*.

Ammonium Oxalate (g/L)	D.M.W (g/L)	Sugar Consumed (g/L)	C.A Produced (g/L)	% Yield
0.00	25.00 ± 4.58 d	60.00 ± 7.75 c	20.50 ± 5.00 b	33 ± 6.08 ab
5.00	27.00 ± 5.20 de	65.00 ± 8.06 d	30.00 ± 6.71 cd	46 ± 7.78 d
10.00	33.00 ± 6.48 e	98.50 ± 9.92 g	77.00 ± 10.00 f	78 ± 8.94 bc
15.00	20.00 ± 3.87 cd	85.00 ± 7.21 f	50.00 ± 8.06 e	58 ± 7.21 e
20.00	15.00 ± 3.46 c	76.00 ± 6.92 e	42.50 ± 7.35 d	55 ± 6.78 de
25.00	12.00 ± 2.87 b	60.00 ± 5.90 c	26.00 ± 5.10 c	43 ± 5.20 c
30.00	11.50 ± 2.65 ab	55.00 ± 5.29 b	23.00 ± 4.69 bc	41 ± 4.98 bc
35.00	10.50 ± 2.30 ab	50.50 ± 4.58 a	20.50 ± 4.00 b	39 ± 4.58 b
40.00	8.50 ± 1.87 a	48.00 ± 4.00 a	15.50 ± 3.46 a	31 ± 3.98 a

Means with different letters within a column are significantly different (*p* < 0.05).

**Table 5:** Optimization of CaCl_2_·2H_2_O concentration for citric acid production by *Aspergillus niger*.

CaCl_2_·2H_2_O (μg/L)	D.M.W (g/L)	Sugar Consumed (g/L)	Citric Acid Produced (g/L)	% Yield
0.00	35.00 ± 5.92 e	99.00 ± 9.95 g	78.00 ± 9.95 g	78.00 ± 9.95 f
0.20	20.00 ± 4.47 d	76.00 ± 8.72 f	45.00 ± 7.35 f	59.00 ± 8.49 ef
0.40	18.00 ± 4.24 cd	70.00 ± 8.37 e	39.00 ± 6.93 e	55.00 ± 7.81 e
0.60	16.50 ± 3.96 c	65.00 ± 7.62 de	29.00 ± 5.39 d	44.00 ± 6.71 d
0.80	14.00 ± 3.74 bc	60.00 ± 7.75 d	25.00 ± 5.00 cd	38.00 ± 6.08 c
1.00	12.50 ± 3.54 b	50.00 ± 7.07 c	15.00 ± 3.87 b	30.00 ± 5.48 b
1.20	10.00 ± 3.16 ab	43.00 ± 6.56 b	22.00 ± 4.69 c	27.00 ± 5.20 ab
1.40	8.50 ± 2.92 a	35.00 ± 5.92 a	8.50 ± 2.92 a	22.00 ± 4.58 a

Means with different letters within a column are significantly different (*p* < 0.05).

## Data Availability

The author confirms that all data generated or analyzed during this study are included in this published article.
